# Musculoskeletal Diseases: From Molecular Basis to Therapy (Volume II)

**DOI:** 10.3390/biomedicines13071722

**Published:** 2025-07-14

**Authors:** Elisa Belluzzi, Assunta Pozzuoli, Pietro Ruggieri

**Affiliations:** 1 Musculoskeletal Pathology and Oncology Laboratory, Department of Surgery, Oncology and Gastroenterology DiSCOG, University of Padova, Via Giustiniani 3, 35128 Padova, Italy; elisa.belluzzi@unipd.it; 2 Orthopedics and Orthopedic Oncology, Department of Surgery, Oncology and Gastroenterology DiSCOG, University of Padova, Via Giustiniani 3, 35128 Padova, Italy; 3 Centre for Mechanics of Biological Materials, University of Padova, 35131 Padova, Italy

Musculoskeletal diseases (MSDs) are among the leading causes of disability worldwide, placing a significant burden on healthcare systems and reducing patients’ quality of life [1]. These disorders include a broad spectrum of conditions such as rheumatological diseases (e.g., osteoarthritis (OA), rheumatoid arthritis (RA), psoriatic arthritis (PsA), and systemic lupus erythematosus (SLE)), muscle and bone disorders, primary musculoskeletal cancers, and cancer-related musculoskeletal complications.

Recent advances in molecular biology, regenerative medicine, and targeted therapies have reshaped both research and clinical strategies in MSDs. Progress in genomics, transcriptomics, and proteomics has enabled the more precise mapping of the signaling pathways involved in the pathogenesis of MSDs. Additionally, the identification of key molecular players, such as cytokines, integrins, matrix metalloproteinases, and microRNAs, has expanded our knowledge and opened new avenues for biomarker discovery and therapeutic target identification.

Despite these advances, significant challenges persist in the diagnosis and treatment of MSDs. Their multifactorial etiology and clinical heterogeneity complicate early detection and personalized treatment strategies. The limited availability of sensitive and specific biomarkers, along with the experimental nature of many emerging therapies, still hinders broad clinical application.

To address these challenges and promote further advancement, we launched a second Special Issue on MSDs, building on the success of the first [2]. This second Special Issue, which was also well received, includes 24 contributions (7 reviews, 1 meta-analysis, and 16 original research articles) and highlights key themes including molecular insights into disease mechanisms and the development of novel therapeutic approaches. The diversity of the published studies reflects the inherently multidisciplinary nature of musculoskeletal research, advancing knowledge from the cellular level upward, encompassing areas such as cell signaling, cell- and biomaterial-based therapies, gene expression and therapy, biomarkers, imaging, and rehabilitation. Collectively, these contributions emphasize the importance of integrating basic science with patient-centered care.

Two reviews, one meta-analysis, and three original articles focus specifically on OA, addressing both its underlying mechanisms and emerging therapeutic strategies. OA, a whole-joint disease, is the most common musculoskeletal disorder, and a leading cause of pain and disability worldwide, particularly among older adults, with no definitive strategies currently available to effectively halt its progression [3]. Although several risk factors have been associated with OA, genetic predisposition remains a key area of active research. In this context, Tyurin et al. (contribution 1) investigated specific genetic markers and identified the Variable Number Tandem Repeat (*VNTR*) locus of the aggrecan gene and the rs7639618 locus of the Double Von Willebrand factor A (*DVWA*) domain-containing protein gene as potential risk factors for knee OA (KOA) in women from the Volga-Ural region of Russia.

Building on the understanding of OA pathogenesis, several contributions highlight innovations in regenerative medicine. Notably, cell-based therapies using mesenchymal stem cells (MSCs), both directly and via secreted exosomes, are being actively explored for tissue regeneration [4,5]. Chu et al. (contribution 2) reviewed the current state of MSC-derived exosomes in OA and their therapeutic potential, emphasizing their ability to modulate the joint microenvironment, reduce inflammation, and promote cartilage repair. Exosomes offer several advantages over traditional and cell-based treatments, including lower immunogenicity and better targeted delivery. However, the need for standardized protocols, deeper mechanistic insights, and rigorous safety evaluations is emphasized.

Similarly, Gan et al. (contribution 3) examined engineered biomaterials such as hydrogels, which reduce joint friction and inflammation. Moreover, these materials are under investigation for localized drug delivery and structural support, offering innovative approaches for OA treatment. Completing these advances, Kim et al. (contribution 4) assessed the effects of integrin αvβ3 on chondrocytes in vitro, showing reduced inflammatory markers, the inhibition of key inflammatory pathways, and the upregulation of the genes involved in chondrogenic and osteogenesis-related factors, suggesting potential for novel targeted therapies.

By translating regenerative approaches into clinical settings, Prizov et al. (contribution 5) investigated the long-term structural changes in the osteochondral unit of 20 patients with KOA who underwent high tibial osteotomy followed by treatment with either platelet-rich plasma or stromal vascular fraction (SVF). Both treatments improved cartilage height and bone architecture, but SVF showed greater regenerative effects, particularly in bone volume, subchondral bone height, trabecular volume, and intertrabecular space, suggesting that it may enhance KOA treatment more effectively.

Addressing the musculoskeletal complications frequently associated with OA, such as sarcopenia in older adults, Lin et al. (contribution 6) conducted a frequentist network meta-analysis of 52 randomized controlled trials (4255 participants) to compare exercise-based interventions. The authors identified that isokinetic training combined with physical modalities and low-load resistance training with blood flow restriction were the most effective strategies for increasing muscle volume and reducing systemic inflammation in individuals with KOA, outperforming monotherapies. Furthermore, the treatment efficacy was influenced by patient age, intervention duration, and follow-up, emphasizing the importance of personalized rehabilitation approaches.

Together, these studies underscore the multifactorial nature of OA and the potential of integrating genetic insights, regenerative therapies, and tailored exercise interventions to improve clinical outcomes in patients with KOA [6].

Knee injuries are recognized as risk factors for OA onset and progression [7,8]. Such injuries could be associated with muscle weakening, particularly of the quadriceps, which exacerbates cartilage damage in the lateral patellofemoral joint. Studnicki et al. (contribution 7) showed that hip manipulation improved vastus medialis activation, thereby improving quadricep balance and helping to prevent joint degeneration.

This Special Issue also provides significant insights into other rheumatologic diseases, such as SLE, a chronic autoimmune and multisystemic disease characterized by a broad spectrum of clinical manifestations and unpredictable disease flares [9]. Fernández-Cladera et al. (contribution 8) examined hematological profiles and found a correlation between the serum complement system and blood cell counts, particularly lymphocytes.

Other contributions focus on RA and PsA, two chronic inflammatory joint diseases with autoimmune components and systemic implications. RA primarily affects the synovial joints and may lead to progressive joint damage and disability, while PsA, often associated with psoriasis, presents with heterogeneous musculoskeletal involvement, including enthesitis, dactylitis, and axial disease [10]. Vlădulescu-Trandafir et al. (contribution 9) analyzed the impact of COVID-19 in RA patients in two Romanian hospitals, revealing more frequent neurological, musculoskeletal, and gastrointestinal symptoms, and increased rates of post-infection flares. Severe cases were linked to age, laboratory markers, and treatments like rituximab, highlighting the need for personalized management. In another study, Pinto-Tasende et al. (contribution 10) found that the elevated synovial expression of IL-17A, Dkk1, and TGF-β1 in early PSA and RA may predict the need for biologic therapies with strong diagnostic accuracy, indicating their potential as early therapeutic biomarkers.

Regarding spine-related MSDs, one study explored how the conditioned medium from intervertebral disk cells inhibits osteogenesis, advancing our understanding of bone regeneration in disk diseases (contribution 11). Research on cervical sagittal balance has also been pivotal in understanding clinical outcomes and the risk of subsidence following anterior cervical discectomy and fusion (contribution 12).

In the field of muscle disorders, three contributions in this Special Issue are specifically dedicated to Duchenne muscular dystrophy (DMD), addressing its systemic complications and exploring novel therapeutic strategies. A narrative review by Cannalire (contribution 13) examined the often-overlooked endocrine and metabolic complications of DMD, including growth retardation, delayed puberty, obesity, insulin resistance, and bone fragility, many of which are exacerbated by prolonged glucocorticoid therapy. The authors emphasized the importance of early monitoring and multidisciplinary care, highlighting the involvement of the GH/IGF-1 axis, altered lipid metabolism, and the RANK/RANKL/OPG pathway as potential therapeutic targets for preserving metabolic and skeletal health. Assefa et al. (contribution 14) reviewed the use of antisense oligonucleotide therapy with Casimersen, approved by the FDA for DMD patients with *DMD* gene mutations amenable to exon 45 skipping, which increases dystrophin production in skeletal muscle. Lin et al. (contribution 15) investigated the Ang/Tie2 signaling pathway in DMD mice’s diaphragm muscle. Interestingly, the authors suggested that increasing the Ang1 concentration and possibly the Ang1/Ang2 ratio may have beneficial effects in promoting mature vascular formation. Given the well-documented vascular abnormalities and impaired perfusion in DMD, enhancing the maturation of blood vessels may represent a critical therapeutic avenue [11]. Additionally, Stratos et al. (contribution 16) assessed calcitriol and Vitamin D receptor modulator 2 (VDRM2) in skeletal muscle regeneration in rats. While VDRM2 showed only moderate effects, the superior outcome with calcitriol underscores the importance of targeting the vitamin D pathway more effectively. In particular, calcitriol improved force recovery, reduced cellular apoptosis, and increased cellular proliferation after muscle injury.

Muscle imbalances, often caused by prolonged static postures, physical inactivity, and overweight, can lead to postural dysfunction and chronic MSDs [12]. Centemeri et al. (contribution 17) found a strong correlation between somatic and postural dysfunctions and MSD risk in adolescents, showing that somatic dysfunctions may represent early warning sings. Early detection and intervention are crucial to prevent progression into chronic conditions that can severely impact quality of life and work performance.

In the context of bone disorders, recent research has explored various strategies to promote bone health and healing. However, a recent systematic review with meta-analysis in humans and rodents revealed that swimming may have different detrimental effects on bone health depending on the anatomical region. In humans, long-term swimming was found to negatively affect cortical bone at the tibial diaphysis, evidenced by decreased cortical thickness alongside compensatory periosteal expansion. Similarly, in animal models, swimming was associated with reduced tibial bone mass without affecting the trabecular microarchitecture. Across both human and animal studies, swimming does not appear to adversely affect the bone mass, cortical geometry, or trabecular architecture in other skeletal sites such as the femur or lumbar spine. Freitas et al. (contribution 18) compared the long-term effects of swimming and running in rats. Their findings showed that swimming induced unbalanced bone turnover, reduced femur growth, lower bone mass, and deterioration in the cortical bone microarchitecture. However, no detrimental effects were observed on the trabecular microarchitecture of the distal femur or the biomechanical properties of the femoral diaphysis. Additionally, no significant differences were found in the circulating bone biomarkers between groups.

Hung et al. (contribution 19) provided a comprehensive narrative review on denosumab, a receptor activator of the nuclear factor kappa-Β ligand (RANKL) inhibitor, highlighting its physiological effects and clinical uses beyond bone remodeling. Denosumab is effective in the long term at preventing fractures, outperforming bisphosphonates in improving bone density, and reducing fracture risk. It also benefits muscle function by increasing the appendicular lean mass and grip strength, thereby lowering fall risk. Emerging evidence also suggests the potential cardiovascular benefits through the positive effects on the cardiac and smooth muscle physiology, and potential reduction in type 2 diabetes risk by improving glucose metabolism. In the context of OA, denosumab has shown promise by suppressing osteoclast activity and preventing chondrocyte apoptosis, thereby potentially slowing disease progression. Despite these multisystem advantages, careful monitoring is essential due to adverse events, including hypocalcemia, jaw osteonecrosis, increased risk of infection, and skin reactions.

In parallel, innovative approaches such as RNA interference (RNAi)-based therapy, which utilizes small interfering RNA (siRNA), are emerging as targeted tools for gene modulation to address the molecular causes of bone degeneration and deformities [13]. Singh et al. (contribution 20) discuss siRNA therapeutics as a promising avenue in bone disease treatment and prevention. Their narrative review also covers various mechanisms for siRNA delivery, and emphasizes the importance of ongoing innovation and collaboration among researchers, clinicians, and industry to drive progress toward clinical application.

Meanwhile, localized antibiotic delivery systems are being investigated as a strategy to manage infected bone defects, which continue to pose a significant clinical challenge due to their complexity and the high risk of treatment failure [14]. The study by Vandenbulcke et al. (contribution 21) illustrated both the promise and limitations of this approach in a rabbit model: while antibiotic-loaded biodegradable scaffolds effectively control infection in segmental bone defects, they may also impede bone union, highlighting a complex trade-off that warrants further investigation.

In cancer-related MSDs, magnetic resonance imaging (MRI) represents a useful tool for detecting soft tissue sarcoma recurrence [15]. The study of Koening et al. (contribution 22) further corroborated the importance of MRI surveillance not only for detecting soft tissue sarcoma recurrence, but also for differentiating recurrence from post-therapeutic changes. Importantly, all local recurrences resembled the primary soft tissue sarcoma, while post-therapeutic changes exhibited distinct characteristics. Bone metastasis poses a major challenge in advanced cancers. Arakil et al. (contribution 23) published a narrative review emphasizing that bone is not just a passive site for metastasis, but actively contributes to cancer progression through its unique microenvironment. The article also reviewed the current diagnostic tools and discussed targeted therapies for pain management and skeletal complications. Finally, it advocated for a multidisciplinary, research-driven approach to improve patient outcomes and care in oncology.

Finally, Touma et al. showed that the exposure of keratinocytes to ultraviolet B (UVB) induced DNA damage, the activation of LINE-1 retrotransposon (the only autonomous retrotransposon still active in the human genome), cellular senescence, and photoaging (contribution 24).

This second Special Issue on MSDs highlights the rapid progress and persistent challenges in understanding and managing these complex conditions. The 24 contributions collectively underscore the multidimensional nature of MSDs—from genetic susceptibility and molecular mechanisms to innovative therapies and personalized rehabilitation. A key theme throughout is the critical role of interdisciplinary collaboration in bridging basic science with clinical and translational research. By highlighting both the complexity and the therapeutic potential of musculoskeletal systems, this Special Issue aims to stimulate further research and inspire novel strategies for preventing and managing these widespread conditions. Looking ahead, integrating molecular diagnostics, regenerative therapies, and digital health tools holds promise for redefining how MSDs are understood and managed. Successfully translating these advances into clinical practice will require sustained collaborative efforts and continued investment in mechanistic research, biomarker discovery, and personalized treatment strategies to ultimately reduce the global burden of MSDs and improve patient outcomes worldwide.
